# Quercetin improves epithelial regeneration from airway basal cells of COPD patients

**DOI:** 10.21203/rs.3.rs-3185241/v1

**Published:** 2023-07-25

**Authors:** Elizabeth S. McCluskey, Nathan Liu, Abhimaneu Pandey, Nathaniel Marchetti, Umadevi Sajjan

**Affiliations:** Temple University; Temple University; Temple University; Temple University; Temple University

**Keywords:** HOXB2, ELF3, goblet cell metaplasia, chronic obstructive pulmonary disease

## Abstract

**Background:**

Airway basal cells from patients with chronic obstructive pulmonary disease (COPD) regenerate abnormal airway epithelium and this was associated with reduced expression of several genes involved in epithelial repair. Quercetin reduces goblet cell metaplasia and the expression of pro-inflammatory cytokines in COPD models. This study assessed whether quercetin improves epithelial regeneration from COPD airway basal cells.

**Methods:**

COPD airway basal cells were treated with DMSO or 1 μM quercetin for three days. The cells were then cultured at air/liquid interface (ALI) for up to 4 weeks. Basal cells from healthy donors cultured at air/liquid interface were used as controls. Polarization of cells was determined at 8 days of ALI. The cell types and IL-8 expression in differentiated cell cultures were quantified by flow cytometry and ELISA. Microarray analysis was conducted on DMSO or quercetin-treated COPD basal cells to identify differentially regulated genes (DEG) and the enriched biological pathways. Bronchial brushings from COPD patients treated with either placebo or quercetin for 6 months were used to confirm the effects of quercetin on gene expression.

**Results:**

Compared to DMSO, quercetin-treated COPD basal cells showed an increase in TER and regenerated the airway epithelium with more ciliated cells, and less goblet cells and IL-8. Comparison of DMSO- and quercetin-treated COPD basal cell transcriptomic profiles indicated that quercetin upregulated genes associated with tissue and epithelial development and differentiation. COPD patients treated with quercetin, but not placebo showed significantly increased expression of two developmental genes HOXB2 and ELF3, which were also increased in quercetin-treated COPD basal cells. Bronchial brushings from active smokers showed significantly increased mRNA expression of TGF-β and IL-8, and it was reduced after quercetin treatment.

**Conclusions:**

These results indicate that quercetin may improve airway epithelial regeneration by increasing the expression of genes involved in epithelial development/differentiation in COPD.

**Trial registration:**

This study was registered at ClinicalTrials.gov on 6–18–2019. The study number is NCT03989271.

## Introduction

Epithelium lining the conductive airways protects the lungs from environmental pollutants and pathogens through mucociliary escalator function and mounting appropriate innate immune responses. Chronic inflammation caused by persistent exposure to environmental insults such as cigarette smoke or other noxious agents may lead to airway epithelial remodeling, which can affect innate immune protective mechanisms. In patients with chronic obstructive pulmonary disease (COPD), airway epithelium often shows basal cell hyperplasia, squamous metaplasia and goblet cell metaplasia and such structural changes affects innate immune responses [1]. Previously, we have shown that in a mouse model of COPD, airway epithelium shows goblet cell metaplasia, which is reduced by oral treatment with quercetin [2, 3].

Airway basal cells of the tracheobronchial tree are specialized tissue-specific stem cells and generate all cell types of the airway epithelium during turnover or repair [1, 4, 5]. Interestingly, epithelium regenerated from airway basal cells isolated from COPD patients shows goblet cell hyperplasia resembling airway epithelium in COPD patients [6–9]. These observations indicate that COPD basal cells may have defects in the repair and regeneration program. Consistent with this, recently we showed that compared to healthy non-smokers airway basal cells from COPD patients show attenuated expression of some transcription factors involved in tissue development and differentiation, which include homeobox (HOX)A1, HOXB2, E74-like ETS transcription factor (ELF3), ELF5 and vestigial like family member (VGLL)1 [10]. The observed differential expression may be due to acquired epigenetic changes which may occur as a result of persistent exposure to inflammatory milieu. Since quercetin reduces airway epithelial remodeling including goblet cell metaplasia in a mouse model of COPD [2, 3], we examined whether quercetin improves airway epithelial regeneration in vitro.

Quercetin (3,3’,4’,5,7-pentahydroxyflavone) is a dietary flavonoid found in many plants. At the molecular level, quercetin scavenges reactive oxidant species thus acting as an antioxidant [11] inhibits various kinases exerting anti-inflammatory effects [12], protects cells from oxidative stress-induced DNA damage [13] and regulates epigenetic changes by modulating DNA methyltransferases, histone deacetylases and long non-coding RNAs [14, 15]. Recently, quercetin was also demonstrated to promote osteogenic differentiation of bone marrow mesenchymal stem cells and epidermal stem cell proliferation by modulating Wnt/β-catenin signaling [16, 17]. Aberrant methylation in airway basal cells has been attributed to abnormal airway epithelial regeneration in COPD [18]. Given the capacity of quercetin in modulating epigenetic changes, we postulated that quercetin may improve regeneration of airway epithelium from basal cells by modulating the genes involved in epithelial cell proliferation and differentiation.

In this study, we show that a brief treatment with quercetin enhances the expression of various developmental genes and promotes polarization and differentiation of basal cells towards ciliated cells. Interestingly, we also demonstrate that bronchial brushings from COPD patients treated with quercetin for 6 months show increased expression of two developmental genes, HOXB2 and ELF3 which may play a role in the polarization and differentiation of airway epithelial cells.

## Methods

### Quercetin:

For cell culture studies, quercetin was kindly provided by Quercegen Pharmaceuticals (Sudbury, MA). HPLC analysis indicated that quercetin was 99% pure. 100mM quercetin was prepared in sterile DMSO and stored in aliquots at −80°C. On the day of use quercetin was dilute to 1 μM in the cell culture medium, filter sterilized prior to use. Similar volume of DMSO diluted in cell culture medium was used as a vehicle control.

For clinical trial, quercetin and placebo formulations were purchased from Nutravail Technologies (Chantily, VA) as soft chews. Each quercetin chew contained 500 mg of quercetin, 350 mg of ascorbic acid and 10 mg of niacin. Each placebo chew had 350 mg of ascorbic acid and 10 mg of niacin.

### Isolation and culturing of airway epithelial cells

Airway basal cells were isolated from bronchial segments of normal donor lungs and explanted lungs from COPD patients at the time of lung transplantation as described previously [6, 19]. The collection of the tissue was approved by the Institutional Review Board of University Michigan, Ann Arbor, MI (HUM00052806) and Temple University, Philadelphia, PA (4407). Patient characteristics are provided in Supplemental Table 1. The basal cells at passage one, were cultured in 6.5 mm collagen-coated transwells as described previously [19, 20]. In some experiments, 80–90% confluent COPD basal cells cultured in 6.5 mm transwells were treated with 0.1, 1 or 5 μM quercetin or equal volume of DMSO (vehicle) for three consecutive days from both apical and basolateral sides. The cells were then harvested for isolation of total RNA or cultured at air/liquid interface (ALI) for up to 4 weeks to promote polarization and mucociliary differentiation of cells.

### Collection of bronchial brushings from COPD patients

The collection of bronchial brushings from COPD patients recruited for clinical trial with quercetin (NCT03989271) was approved by Temple IRB #25738. Patient characteristics are presented in Supplemental Table 2. In this clinical trial, patients were treated with a placebo or 2000 mg/day quercetin for 6 months. Post-bronchodilator FEV1 was measured and bronchial brushings collected from 4 placebo and 7 quercetin-treated COPD patients at baseline and at the end of the treatment. The cells from the bronchial brushings were harvested by centrifugation, lysed in TRIZOL and stored at −80° C. The total RNA was isolated from all the samples at the same time. Blood was collected and analyzed for quercetin levels by reverse phase HPLC as described previously [21]. Blood quercetin levels at baseline was comparable between placebo (0.153 ± 0.086 μM) and quercetin group (0.195 ± 0.069 μM). Six months after treatment, while placebo-treated patients showed no significant increase, quercetin-treated patients showed 11fold increase in their blood quercetin levels.

### Transepithelial resistance.

Transepithelial resistance (TER) was measured using the EVOM voltmeter equipped with ENDOHM-6 EVOM electrode (World Precision Instruments, Sarasota, FL) and the TER was expressed as Ohms/cm^2^.

### ELISA

After culturing the cells for 4 weeks at air/liquid interface, transwells were transferred to a new receiver plate containing fresh medium and incubated for 24 h. The basolateral medium was collected and IL-6 and IL-8 protein levels were measured by ELISA (R & D systems, Minneapolis, MN).

### Total RNA isolation

Total RNA was isolated from TRIZOL lysates of normal basal cells, DMSO and quercetin-treated COPD cells, and bronchial brushings using a Direct-zol^™^ miniprep kit (Zymo research, Irvine, CA). The integrity of total RNA was determined by Agilent 2100 bioanalyzer and the RNA integrity number was consistently >7.

### Microarray processing

Biotinylated cDNAs synthesized from total RNA isolated from DMSO- or quercetin-treated COPD basal cells were subjected to microarray analysis using Human Gene 2.1 ST arrays. To identify statistically significant differential gene regulation (p-value of < 0.05 and up/down regulated by more than 2-fold), we performed pairwise comparison (DMSO versus quercetin) by the LIMMA methodology (Linear Models for Microarray Data). Normalized data and raw data are available in Gene Expression Omnibus (GEO) with accession number GSE137557.

### Gene Ontology

Gene Ontology (GO) and KEGG pathways were analyzed with WebGestalt (WEB-based GEne SeT AnaLysis Toolkit) [22] using the Benjamini-Hochberg correction for multiple testing (FDR 5%). For Gene Ontology, only Biological Process terms are discussed because Cellular Component and Molecular Function terms were less relevant.

### Flow cytometry

Mucociliary-differentiated cells were dissociated with accutase (ThermoFisher Scientific, Waltham. MA), cells were fixed with 4% paraformaldehyde, and then blocked/permeabilized by incubating in PBS containing 1% BSA and 0.5% saponin for 30 min. Cells were then incubated with acetylated tubulin (cat#T7451, Sigma Aldrich, St. Louis, MO), Muc5AC (cat#ab24071TP63, Abcam, Cambridge, MA) or TP63 (Cat#ab32353, Abcam), and bound antibodies were detected by using Alexafluor-labeled second antibodies as previously described [10]. The cells were then analyzed in FACS Calibur Flow cytometer (BD Biosciences, San Jose, CA), and data was analyzed by FlowJO version 10 (Tree Star, Ashland, OR).

### Histology

For histological evaluation, cell cultures were fixed in buffered formalin, embedded in paraffin, 5μ thick sections were deparaffinized and stained with hematoxylin and eosin (H and E) or periodic acid Schiff’s (PAS) reagent.

### Real-time PCR

cDNA was synthesized from total RNA (High-capacity first strand synthesis kit, Thermofisher Scientific) and subjected to qPCR to determine the expression of *ELF5, ELF3, GRHL1, IVL, WNT5, ZO-3, HOXA1, HOXB2, VGLL1, IL-8, TGF-β and glyceraldehyde 3-phosphate dehydrogenase* (*GAPDH*) using gene-specific primers and probes. The expression level of each gene is presented as a fold change over the house-keeping gene, *GAPDH*.

### Statistical analysis

Data are presented as mean ± SD or median with range. Statistical significance was assessed by t test or Mann-Whitney test to compare two groups and ANOVA or ANOVA on Ranks with Kruskal Wallace nonparametric test to compare three groups as appropriate. For pairwise comparison, a signed rank test was used. For clinical samples, due to high variability in the gene expression at baseline, we compared the gene expression at base line and after completion of treatment for each patient and the statistical significance was determined by Shaipiro-Wilk T test. A p-value of ≤0.05 was considered statistically significant.

## RESULTS

### Quercetin improves airway epithelial regeneration from COPD basal cells.

Polarization is an important early step during the regeneration of airway epithelium from airway basal cells. Previously, we have demonstrated that compared to normal, COPD basal cells cultured at ALI have lower TER [10]. Therefore, we examined whether quercetin increases TER in COPD cells. In our initial study, airway basal cells from one COPD patient was used to find an optimal concentration of quercetin in improving TER. Ninety percent confluent monolayer of COPD basal cells was treated with 0.1, 1, or 5 μM quercetin or DMSO (vehicle) for 3 days, and cells were cultured at ALI without quercetin up to 10 days. The TER was measured at 4, 6, 8 and 10 days. Normal basal cells were cultured similarly without any treatment and used as positive control showed maximum TER on day 8 which did not change at 10 days (Supplemental Figure 1). As observed previously, untreated basal COPD basal cells showed reduced TER compared to normal basal cells. Treatment with DMSO or 0.1 μM quercetin had no effect on TER of COPD basal cells. On the other hand, cells treated with 1 μM quercetin showed increase in TER and was almost equivalent to normal basal cells. Cells treated with 5 μM quercetin showed some cell death during treatment and therefore the TER was not measured. Based on these results, we choose to use quercetin at 1 μM and to measure TER after 8 days of culturing the cells at ALI in the subsequent experiments.

**Figure 1:**
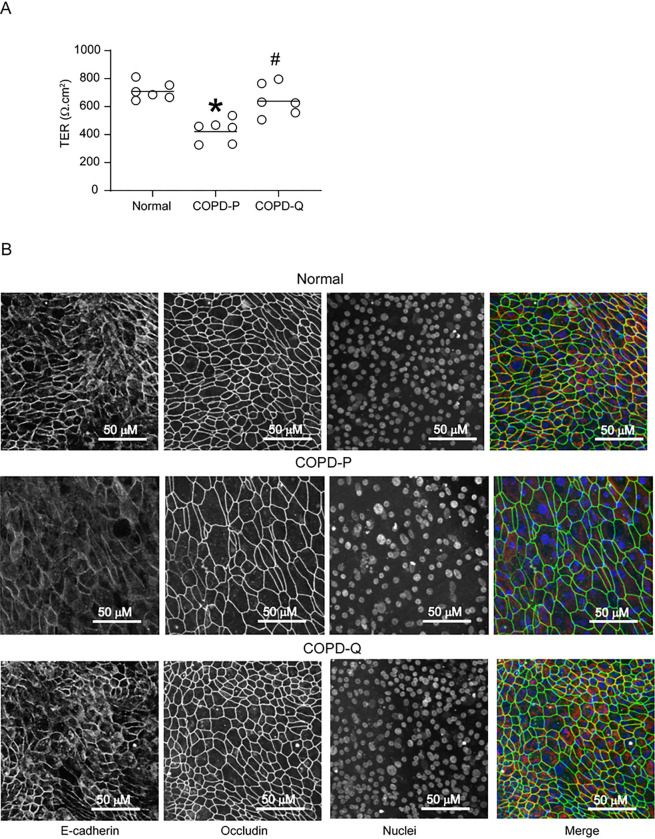
COPD basal cells treated with quercetin show improvement in developing intercellular junctions. A. Ninety percent confluent monolayer of COPD basal cells treated with DMSO (COPD-P) or 1 μM quercetin (COPD-Q), and normal basal cells were cultured at air/liquid interface for 8 days. A. The TER was measured and the data was expressed as range with median (n=6, ANOVA on ranks, *p≤0.05, different from normal; # p≤0.05, different from COPD-P). B. Cultures were fixed in cold methanol, blocked with BSA and incubated with antibody to occludin and E-cadherin and the bound antibodies were detected by antimouse IgG conjugated with AlexaFluor 488 (occludin) and antirabbit IgG conjugated with AlexaFluor 594. The nuclei were counterstained with DAPI and the cells were imaged using confocal microscopy. Images representative of 3 experiments.

Airway basal cells from COPD patients were cultured in transwells until they reached 90% confluency. Cells were then treated with 1 μM quercetin or DMSO for 3 consecutive days. The cells were then cultured at an ALI for up to 4 weeks without quercetin to promote polarization and differentiation into mucociliary phenotype. Cell polarization was determined by measuring TER 8 days after shifting the cells to an ALI and compared with the TER of normal basal cells. Compared to placebo, COPD basal cells treated with quercetin showed significantly higher TER and was comparable to TER of healthy non-smokers’ basal cells (Figure 1A). Quercetin-treated COPD cells also showed increased localization of E-cadherin and occludin to the intercellular junctions compared to placebo-treated cells (Figure 1B).

**Figure 2:**
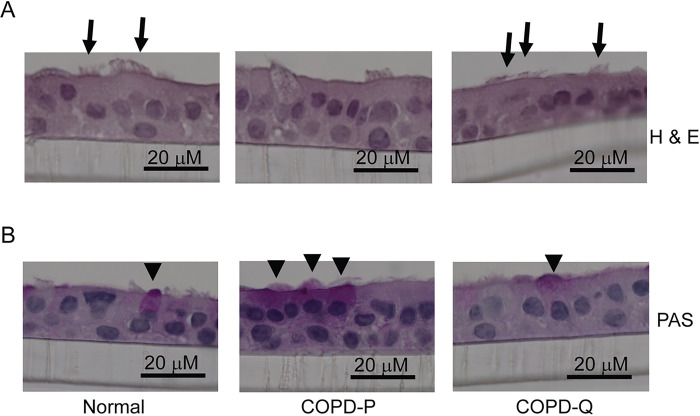
Quercetin treatment improves differentiation of COPD basal cells. Ninety percent confluent monolayer of COPD basal cells treated with DMSO (COPD-P) or quercetin (COPD-Q), and normal basal cells were cultured at air/liquid interface for 4 weeks. The cultures were fixed in 10% buffered formalin, embedded in paraffin. Five micron thick sections were deparaffinized and stained with H & E or PAS. The images are representative of 3 experiments.

Differentiated cell cultures from quercetin-treated basal cells showed more ciliated cells and fewer goblet cells by histology (Figure 2A and 2B). Flow cytometric analysis indicated an increase in the number of ciliated cells and a decrease in goblet and basal cells in quercetin-treated cells when compared to placebo-treated cells (Table 1). These results indicate that quercetin may improve airway epithelial cell polarization and differentiation.

### Quercetin treatment reduces pro-inflammatory phenotype in COPD cell cultures.

**Figure 3:**
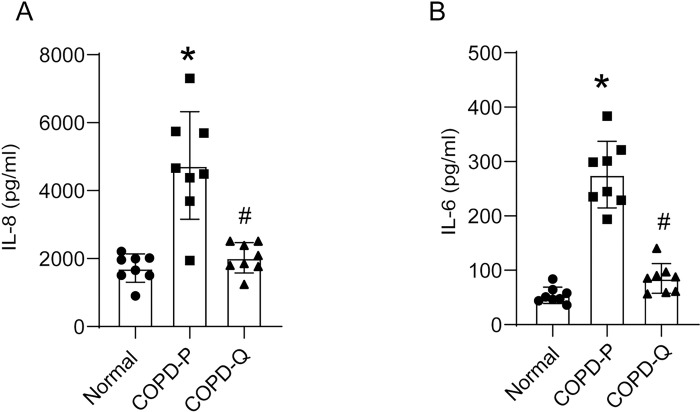
Mucociliary-differentiated cell cultures established from quercetin-treated COPD airway basal cells show attenuated levels IL-8 and IL-6. Ninety percent confluent monolayer of COPD basal cells treated with DMSO (COPD-P) or quercetin (COPD-Q), and normal basal cells were cultured at air/liquid interface for 4 weeks. The transwells were transferred to new receiver plates containing fresh medium and incubated for 24 h. Basolateral medium was collected and levels of IL-8 and IL-6 were measured by ELISA. Data represent mean ± SD (n=8, ANOVA, *p≤0.05, different from normal; # p≤0.05, different from COPD-P).

Mucociliary-differentiated COPD cell cultures produced higher than normal levels of IL-8 and IL-6 proteins as observed previously [6]. The differentiated airway epithelial cell cultures generated from quercetin-, but not placebo-treated COPD basal cells showed reduction in the levels of both IL-6 and IL-8 (Figure 3).

### Quercetin treatment reduces pro-inflammatory phenotype in COPD cell cultures.

To identify the possible pathways that are altered by quercetin in COPD airway basal cells we conducted microarray analysis on placebo- and quercetin-treated COPD basal cells from 8 donors. In a pairwise comparison of placebo vs quercetin, 1527 genes showed differential expression, 673 mapping to upregulated genes and 854 mapping to downregulated genes in quercetin-treated COPD cells (Supplemental Figure 2 and Supplemental Table 3).

Gene ontology (GO) analysis of the upregulated genes in quercetin-treated COPD cells showed an overrepresentation of genes associated with epithelium and tissue development, and epithelial and epidermal differentiation (Table 2). Epithelial development and differentiation categories included genes encoding keratins, ELF3, ELF5, grainyhead like transcription factor (GRHL)1, GRHL3, Kruppel-like transcription factor (KLF) 4, desmoglein (DSG) 1, S100 calcium binding protein-A7 (S100-A7), bone morphogenetic protein 2, frizzled B, Wnt5A, uroplakin1A, filaggrin (FLA), involucrin and tight junction protein ZO3 (TJP3). GO analysis of down regulated genes by quercetin showed over representation of genes associated with cell cycle inhibition (Supplemental Table 4). These microarray results indicate that quercetin treatment may improve the polarization and differentiation of COPD basal cells by positively regulating pathways involved in polarization and differentiation and inhibiting cell proliferation.

### Validation of upregulated genes by quercetin in COPD cells

**Figure 4:**
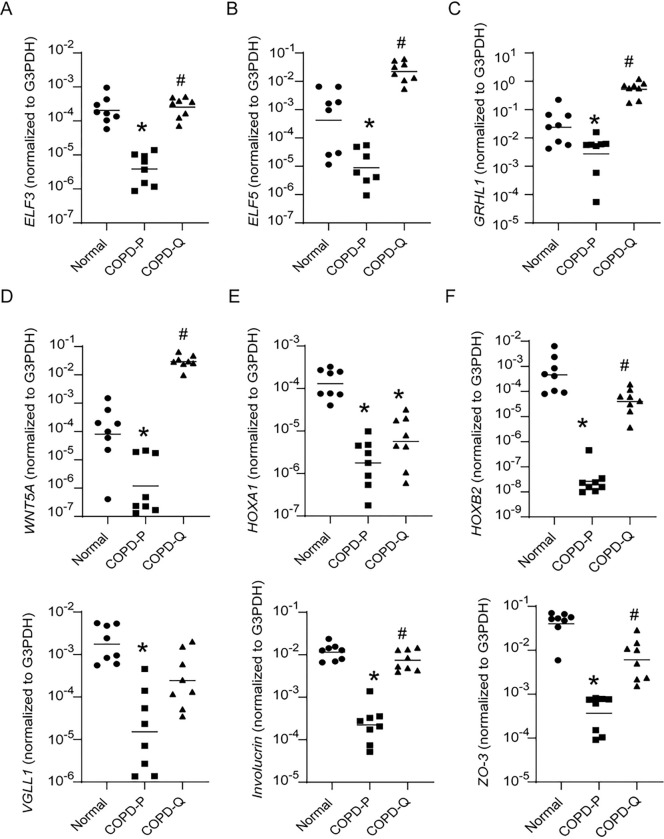
Quercetin treatment enhances expression of developmental genes. Total RNA was isolated from 90% confluent monolayer of COPD basal cells treated with DMSO (COPD-P) or quercetin (COPD-Q), and normal basal cells. cDNA was synthesized and mRNA expression of developmental genes, involucrin, ZO-3 and G3PDH was determined by qPCR. The data was expressed as fold change over G3PDH and presented as range with median (n=8, ANOVA on ranks, *p≤0.05, different from normal; # p≤0.05, different from COPD-P).

RT-qPCR was conducted to validate the results of microarray analysis. We measured the expression of a few of the upregulated genes involved in epithelial cell development and differentiation by a probe-based PCR. Compared to placebo-, quercetin-treated cells showed significant increases in the expression of ELF3, ELF5, GRHL1, WNT5A, Frizzled B and tight junction protein ZO3 (TJP3), all of which were reduced in COPD compared to normal (Figure 4). We also measured the expression of HOXA1, HOXB2, VGLL1 which we had demonstrated previously to be reduced in COPD compared to normal basal cells (Figure 4). Compared to placebo, quercetin-treated cells showed increased expression of HOXB2, but not HOXA1 and VGLL1.

### Bronchial brushings from COPD patients treated with quercetin show increased expression of genes involved in epithelial cell development and differentiation

**Figure 5:**
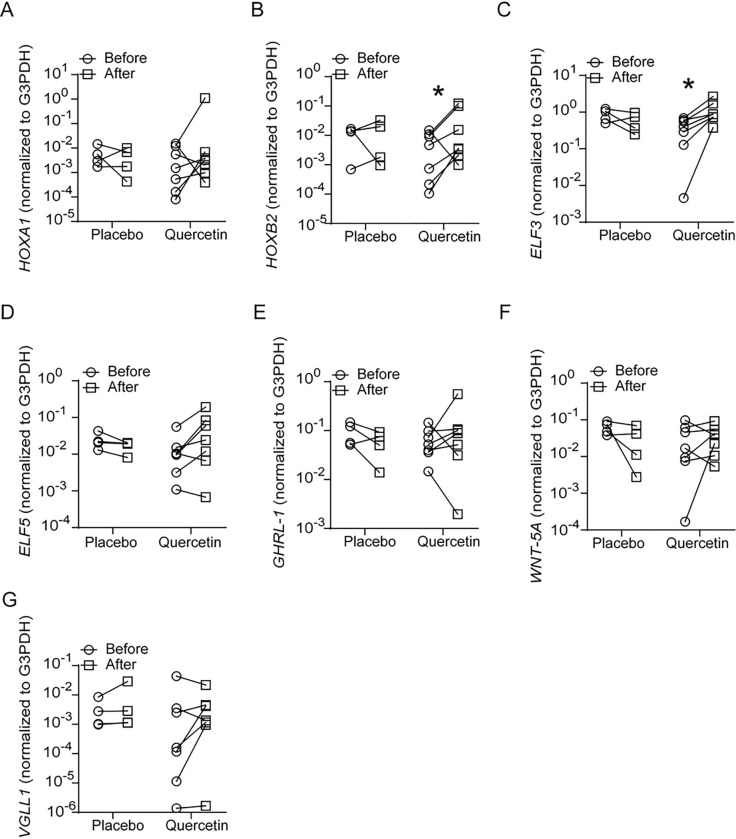
COPD patients treated with quercetin show increased expression of HOXB2 and ELF3. COPD patients were treated with placebo (4 patients) or querecetin (7 patients) for 6 months. Total RNA isolated from bronchial brushings obtained before and after completion of treatment was subjected RT-qPCR with gene-specific primers and probe. The data was expressed as fold increase over G3PDH and represents intracomparison of gene expression before and after treatment with placebo or quercetin (#p ≤ 0.05, different from sham, paired Shaipiro-Wilk T test).

**Figure 6:**
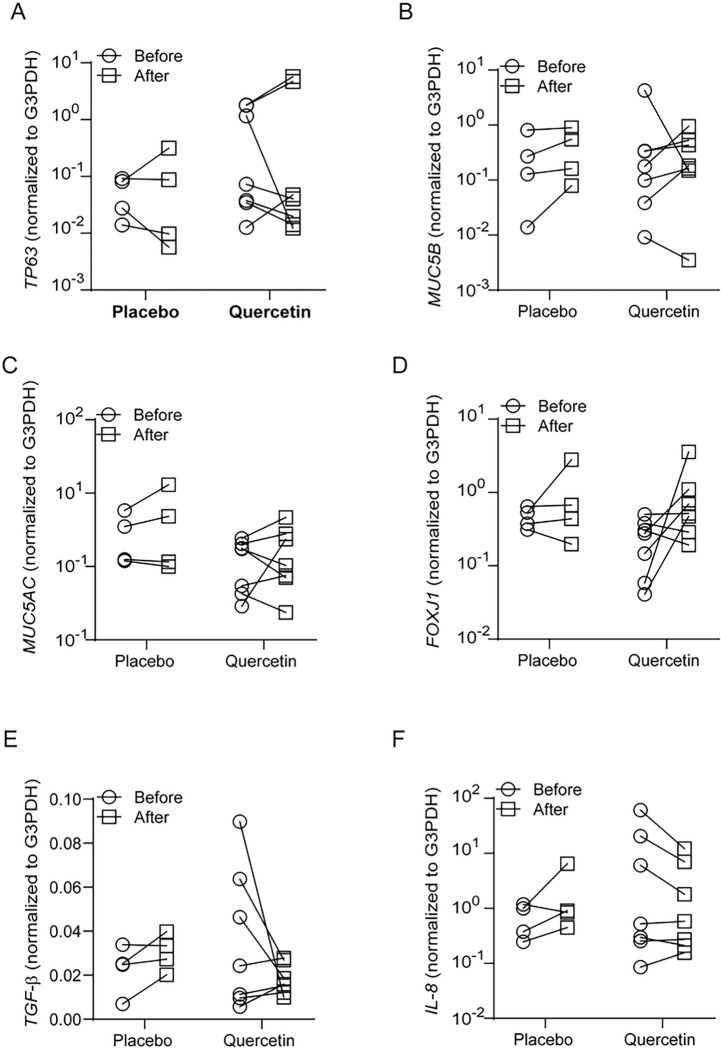
Effect of quercetin on the markers of different cell types and cytokines. COPD patients were treated with placebo (4 patients) or querecetin (7 patients) for 6 months. Total RNA isolated from bronchial brushings obtained prior to and after completion of treatment was subjected RT-qPCR with gene-specific primers. The data was expressed as fold increase over G3PDH and represents intracomparison of gene expression before and after treatment with placebo or quercetin. Statistical significance was analyzed by paired Shaipiro-Wilk T test.

Recently, we completed a small placebo-controlled clinical trial with quercetin in COPD patients. In this clinical trial, we had 4 patients treated with placebo and 7 with 2000 mg/day quercetin for 6 months. Plasma quercetin levels at baseline ranged between 0.33 to 0.63 μM with an average of 0.467 ± 0.104 μM. Quercetin, but not placebo-treated patients showed 1.4 to 4.9 fold increase in plasma quercetin levels over their respective baseline at the end of treatment period. FEV1 % predicted did not change significantly in either placebo or quercetin group. From these studies, we also had access to bronchial brushings collected at baseline and at the end of treatment. Total RNA was isolated from the bronchial brushings and analyzed for the expression of HOXA1, HOXB2, VGLL1, ELF3, ELF5, GRHL1, and WNT5A, and also markers of different cell types of airway epithelium, such as TP63 (basal cell marker), FOXJ1 (ciliated cell marker), and MUC5B and MUC5AC (goblet cell markers). Out of these genes, the expression of only HOXB2 and ELF3 showed a significant increase in quercetin-, but not in placebo-treated patients (Figure 5). Three out of 7 quercetin-treated patients also showed an increase in HOXA1 and VGLL1 expression. There was no alteration in the expression of mucin genes or basal cell marker TP63 in both placebo- and quercetin-treated patients (Figure 6A–6C). The expression of FOXJ1 increased in 4 out of 7 quercetin-treated patients and one out of 4 placebo-treated patients (Figure 6D).

Next, we examined the expression of TGF-β, which contributes to persistent epithelial to mesenchymal transition and abnormal repair in COPD [8], and the expression of pro-inflammatory cytokines, IL-6 and IL-8. Placebo-treated patients showed no alteration in the expression of either TGF-β or IL-8. IL-6 was below the detection limit in both placebo-and quercetin-treated patients. Interestingly, four out of 7 quercetin-treated patients showed a reduction in the mRNA expression of TGF-β (Figure 6E and 6F). Three out of these 4 patients also showed reduced expression of IL-8 mRNA. Together, these results indicate that quercetin may promote normal repair/regeneration of airway epithelium in COPD by inducing the expression of developmental genes.

## Discussion

The present study shows that treatment of COPD airway basal cells with quercetin corrects defects in airway epithelial regeneration and this was associated with upregulation in the expression of several developmental genes that may participate in epidermal and epithelial differentiation. Interestingly, we also show that two of the developmental genes, HOXB2 and ELF3 which increased in COPD basal cells after treatment with quercetin *in vitro*, were also significantly increased in COPD patients treated with 2000 mg/day quercetin for 6 months.

Human airway basal cells from tracheobronchial tissue regenerate epithelium that resembles airway epithelium in vivo. The airway basal cells express several developmental genes including ELF3, ELF5, WNT5A, NOTCH1–4, GRHL1, GRHL2 and VGLL1. Out of these genes, WNT, NOTCH, GRHL2 and ELF3 have been demonstrated to play a role in bronchial epithelial regeneration and maintaining homeostasis [23–26]. COPD basal cells regenerate abnormal airway epithelium showing goblet cell hyperplasia and pro-inflammatory type [6, 27]. This was associated with reduced expression of several of these developmental genes and also previously unidentified developmental genes HOXA1 and HOXB2 [10]. In the present study, we found that a brief treatment with quercetin significantly increases the expression of developmental genes including HOXB2, ELF3, ELF5, GRHL1, GRHL2, WNT5A, and VGLL1 and this was associated with improved polarization and differentiation.

Previously, we have shown that knockdown of HOXB2 contributes to polarization of airway epithelial cells [10]. There are also reports demonstrating that knockdown of ELF3 resulted in delayed repair of bronchiolar epithelium following injury in mice and also a reduction in the expression of TGF-β type II receptor, which is involved in epithelial cell differentiation indicating ELF3 may be required for both airway basal cell proliferation and differentiation [23]. Genetic inhibition of GRHL2 in airway epithelial cells reduced the expression of E-cadherin and claudin 4. Therefore, it is possible that GRHL2 may play a role in the development of tight junctions [28]. Canonical activation of WNT/β-catenin signaling not only maintains the stemminess of the basal cells during the proliferation of basal cells but also cilia formation in multiciliated cells during differentiation [25]. Based on this literature, it is reasonable to presume several developmental genes function in a coordinated fashion to regenerate airway epithelium. Quercetin may improve airway epithelium regeneration by increasing the expression of several developmental genes in COPD basal cells.

COPD is not a genetic disorder, therefore, defect in the expression of these developmental genes may be due to epigenetic changes that occurs as a result of chronic exposure to an inflammatory milieu. Consistent with this notion, abnormal airway epithelial regeneration was attributed to aberrant methylation in airway basal cells in COPD [18]. Since quercetin has an ability to modulate epigenetic changes by decreasing the activities of DNA methyltransferases, histone deacetylases and histone methyltransferases [29], we speculate that quercetin may enhance the expression of developmental genes by erasing the acquired epigenetic changes in COPD basal cells.

Interestingly, we also noted negative regulation of genes that are associated with cell cycle and proliferation in quercetin-treated COPD basal cells. Inhibition of cell cycle and proliferation may be necessary during the repair process after the cells reach confluency (which represent wound closure following injury) to promote polarization and differentiation. If the proliferation of basal cells continues after the wound closure, it can lead to basal cell hyperplasia, which is often observed in patients with COPD [4]. In addition, it may also lead to persistent EMT and abnormal differentiation resulting in goblet cell and squamous metaplasia.

Intriguingly, we observed that treatment with quercetin, but not placebo for 6 months significantly upregulated the expression of HOXB2 and ELF3 in the bronchial epithelial cells of COPD patients. Additionally, 3 out of 7 quercetin-treated patients also showed increased expression of ciliated cell marker, FOXJ1. Interestingly, these same three patients also showed 65 to 80% and 60 to 90% reduction in the expression of TGF-β and and IL-8 respectively. All these three patients are current smokers and show higher basal levels of TGF-β and and IL-8. Cigarette smoke enhances TGF-β expression, which drives epithelial to mesenchymal transition [8]. Sustained epithelial to mesenchymal transition impair regeneration of airway epithelium and enhance expression of pro-inflammatory cytokines [30]. HOXB2, which promotes polarization [31] and ELF3, which represses epithelial to mesenchymal transition and participates in bronchial epithelial repair [23, 32] together may improve polarization and repair of bronchial epithelium, which in turn may promote normal differentiation of cells and reduce the expression of TGF-β and IL-8.

There are some limitations with this study. The COPD basal cells used for microarray analysis are from patients with end-stage disease and therefore may show increased dysregulation in the expression of genes involved in repair and regeneration of airway epithelium. Therefore, we expect that airway basal cells from COPD patients with mild to moderate lung disease may show variable changes in the expression of these developmental genes, which will be a subject for future investigation. Second, there were very few patients in each group and is necessary to confirm these findings in a larger group of patients with different disease severity. Third, we utilized bronchial brushings which will have a mixture of ciliated, goblet and basal cells along with other minor cell types of bronchial epithelium to determine the quercetin-induced expression of developmental genes. Since basal cells are tissue specific stem cells, we expect these cells to express higher levels of developmental genes than other cell types of bronchial epithelium. Therefore, we may observe more robust quercetin-induced changes in the isolated basal cells from bronchial brushings.

In summary, our results suggest that quercetin may promote normal airway epithelium regeneration from COPD basal cells by modulating the expression of developmental genes, particularly, HOXB2 and ELF3. In the future studies, we will focus on the elucidating the mechanisms by which these two developmental genes coordinate to promote polarization and differentiation of airway epithelium in COPD.

## Figures and Tables

**Table 1.** Quantitation of ciliated, goblet and basal cells in COPD cell cultures

Cell type	Normal	COPD-P	COPD-Q
Ciliated cells	44.98 ± 2.49#	26.49 ± 10.46*	34.53 ± 7.13#
Goblet cells	15.53 ± 2.84#	26.88 ± 6.93*	19.71 ± 4.36#
Basal cells	23.36 ± 2.86	33.00 ± 7.77*	28.46 ± 2.48

The data represent mean ± SD (n=6 subjects in each group);

* different from normal;

# different from COPD-P

**Table 2.** Top 10 enriched Biological Process terms for upregulated genes in COPD-Q group

Gene Set	Description	Size	Expect	Ratio	P Value	FDR
GO:0008544	Epidermis development	437	11.456	4.2774	<2.2e-16	<2.2e-16
GO:0070268	Cornification	106	2.7787	9.3569	<2.2e-16	<2.2e-16
GO:0043588	Skin development	390	10.224	4.3038	2.2204e-16	6.6251e-13
GO:0030216	Keratinocyte differentiation	280	7.3400	5.0409	4.4409e-16	9.9376e-13
GO:0009913	Epidermal cell differentiation	331	8.6769	4.6100	5.5511e-16	9.9376e-13
GO:0031424	Keratinization	210	5.5050	5.6313	4.8850e-15	7.2876e-12
GO:0030855	Epithelial cell differentiation	712	18.664	3.0539	3.9524e-14	5.0540e-11
GO:0060429	Epithelium development	1166	30.566	2.2247	2.9774e-10	2.9612e-7
GO:0009888	Tissue development	1839	48.208	1.9084	4.7829e-10	4.2812e-7

## Data Availability

All the data are available are presented in the manuscript. The microarray data has been deposited into public domain.
